# Supplementary figures and images for: NIRis: A low-cost, versatile imaging system for near-infrared fluorescence detection of phototrophic cell colonies used in research and education
Source: PLoS One. 2024 May 21;19(5):e0287088. doi: 10.1371/journal.pone.0287088 (PMC11108223; doi:10.1371/journal.pone.0287088)

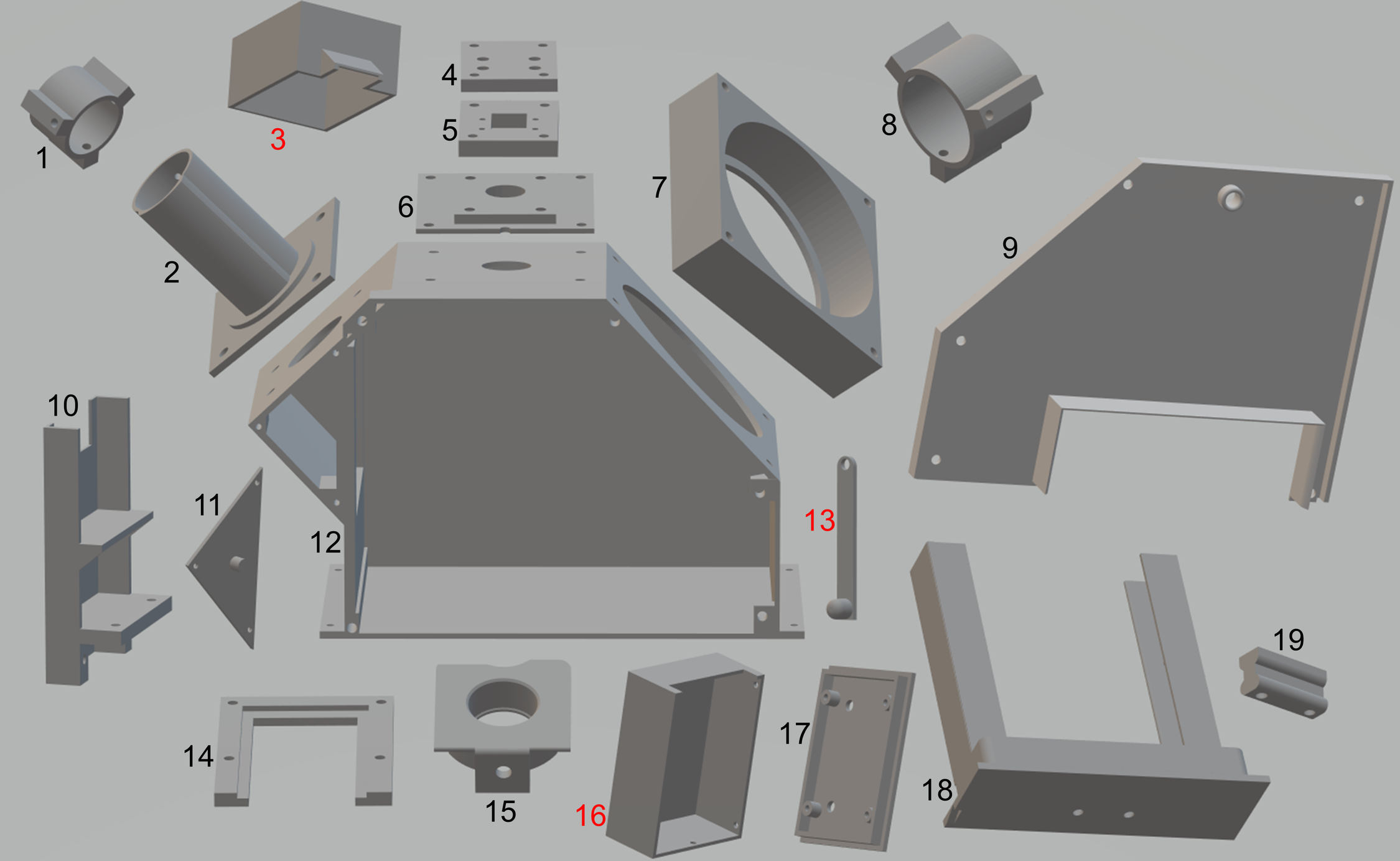

Supplement: S1 Fig — Parts are rotated to display their construction. Parts with numbers in red are optional. 1 & 8: Flashlight caps to access the switches. 2 & 7: Flashlight holders 3: Camera assembly cover 4–6: Camera assembly parts. Camera module is placed between part 5 & 6. 9 & 11: Front wall 10: Raspberry Pi holder to be mounted to the back. 12: Main chamber 13: Security clamp (2X), can be mounted in two positions in front and on top to prevent Raspberry Pi and the sample drawer from sliding out. 14: Mount for filter holder—to be fixed underneath the top opening. 15: Filter holder, slides into mount (14). A bolt is secured in the front hole reaching through the corresponding hole of the front plate so that the filter holder can be engaged and disengaged 16: Cover for the relay board. 17: Base plate holding the relay board—to be fixed at the back. 18: Sample drawer for Petri dishes 19: Handle for the sample drawer. (TIF) [file pone.0287088.s001.tif]

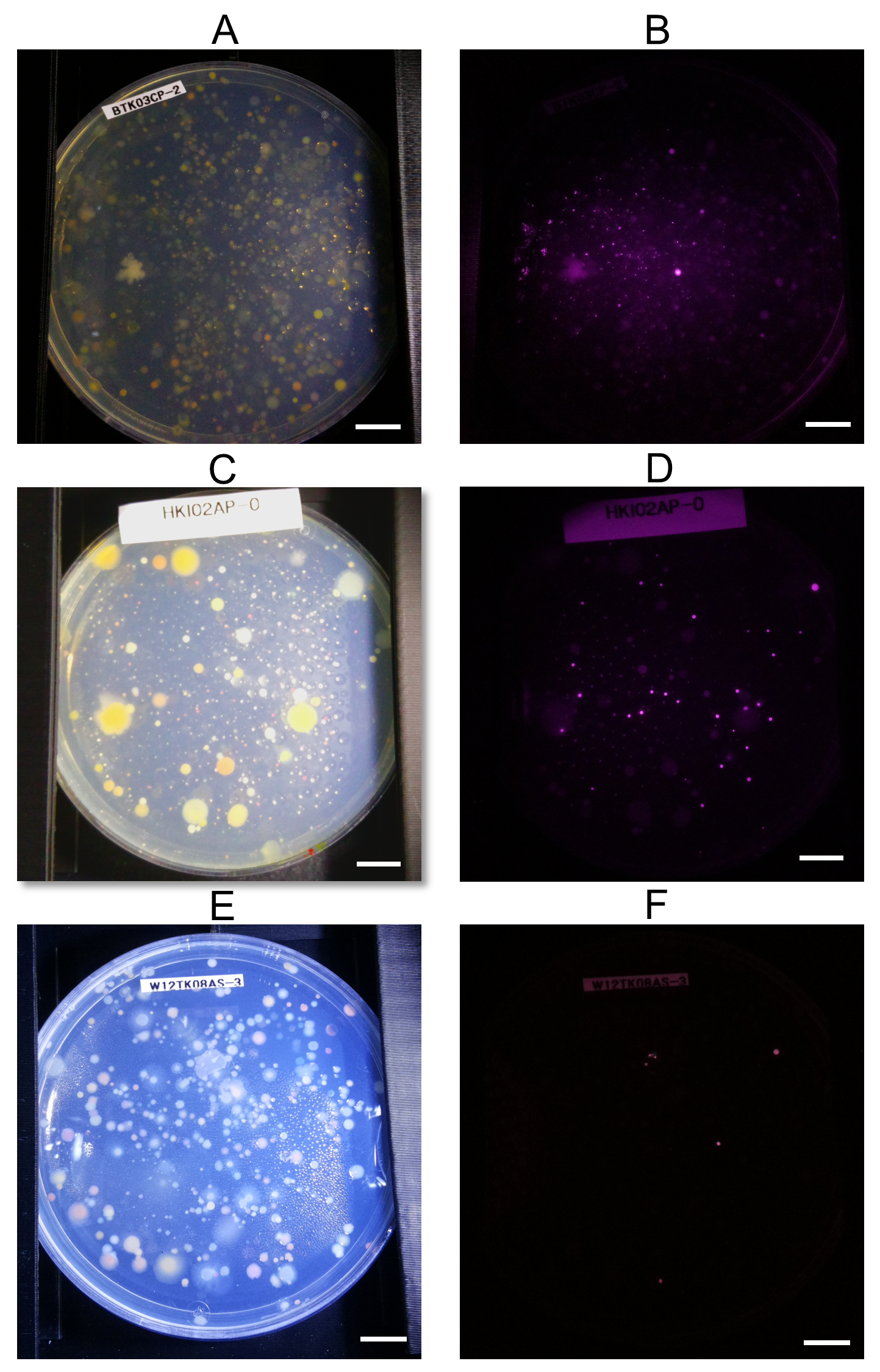

Supplement: S2 Fig — A & B: White light and fluorescence images taken with an early iteration of NIRis, which had a weaker 12 LED, non-diffused excitation light and a single excitation filter. The reliable identification of fluorescent colonies across the entire plate is hard. Reliable automation of the AAPB identification would be impossible. C & D: White light and fluorescence images with NIRis which employed a sufficiently diffused and weaker 12 LED light source with a single excitation filter. Reflections of water droplets on the lid and negative colonies bleed through making it difficult to automate the identification with software. E & F: Current set-up of NIRis with a strong 128 LED excitation lamp and two stacked excitation filters. Even though the sample has large white colonies and water droplets on the lid, only truly fluorescent colonies are visible in the fluorescent mode. Scale bars = 1 cm. (TIF) [file pone.0287088.s002.tif]

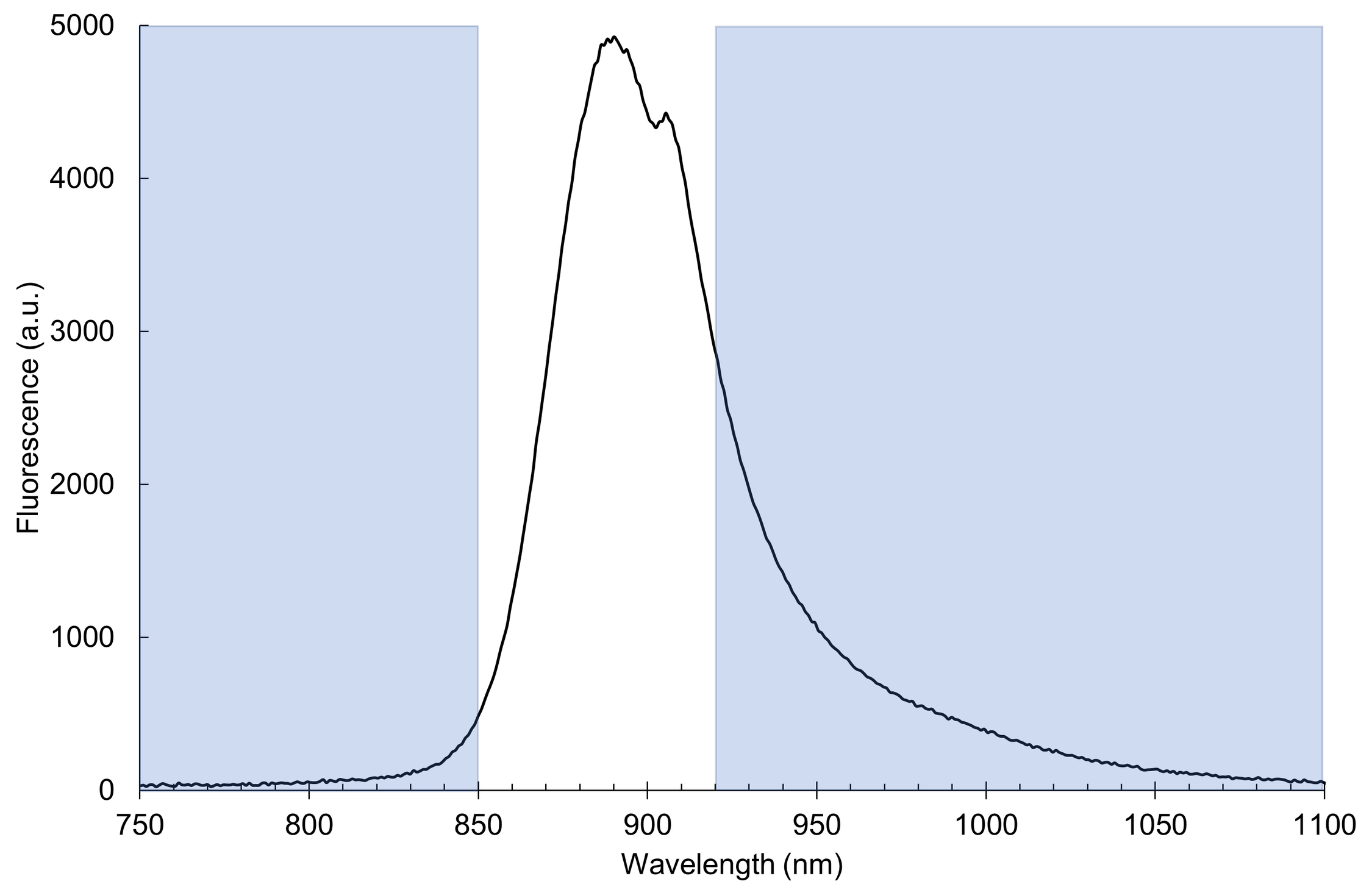

Supplement: S3 Fig — An UV-induced fluorescence spectrum recorded from Sphingomonas glacialis strain S2U11 showing the approximate wavelength range passing through the 880 nm ± 8 nm bandpass filter (FWHM = 70 ± 8 nm) to the sensor (white area). The imaging set-up combined the same excitation flashlight rated at 395 nm used in NIRis with a NIR-sensitive iDus InGaAs Spectroscopy CCD Camera (Andor Technology, Belfast, Ireland) as described in [12]. (TIF) [file pone.0287088.s003.tif]

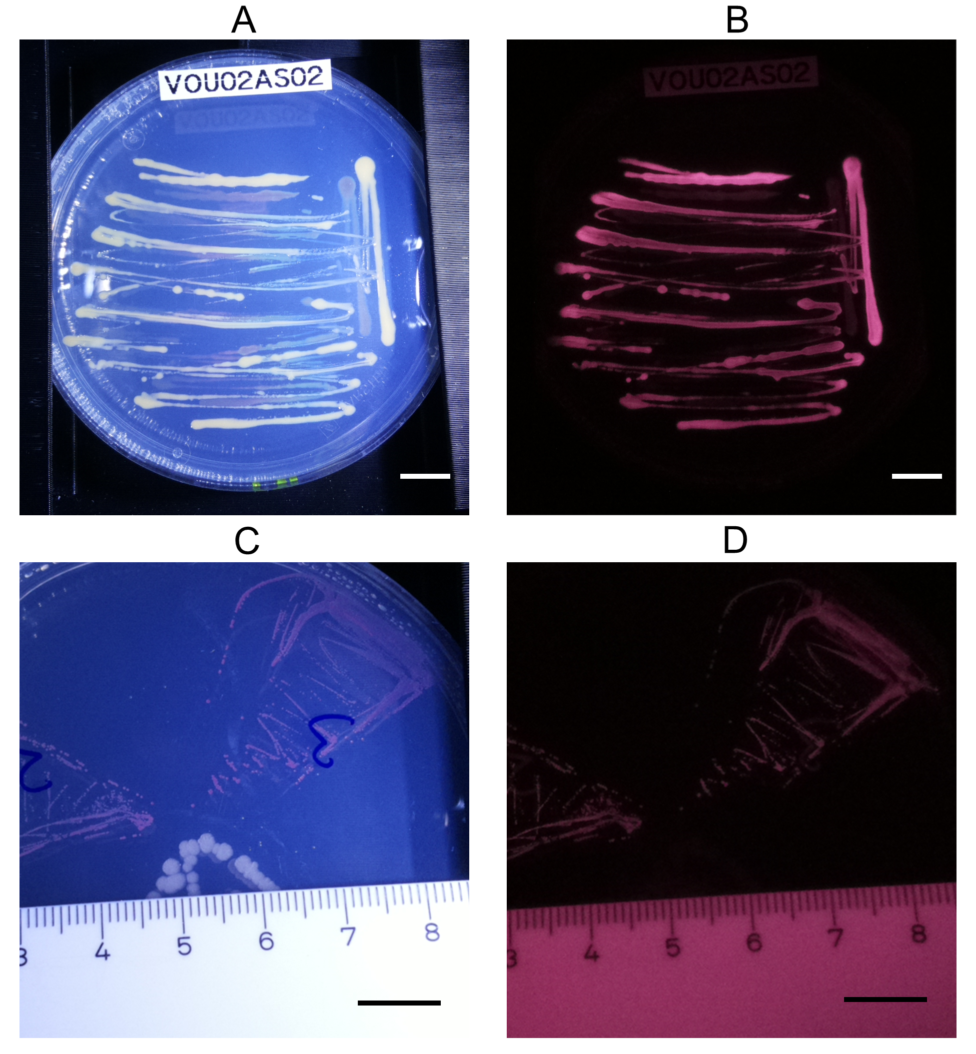

Supplement: S4 Fig — A: White light reference image of a strain of Sphingomonas faeni streaked out for fluorescence confirmation. B: The UV-induced NIR fluorescence image of the same Petri dish as in A, showing a strong fluorescence of the strain. C: A magnified white light reference image of three unidentified isolates, together with a regular ruler with millimeter graduation to emphasize the scale. D: The same Petri dish and frame as in C but imaged in the fluorescence mode. Both pink strains are NIR fluorescent, the white strain is not. The diameters of the smallest positive colonies are clearly below 0.5 mm. Scale bars = 1 cm. (TIF) [file pone.0287088.s004.tif]
