# Supplementary material for: NIRis: A low-cost, versatile imaging system for near-infrared fluorescence detection of phototrophic cell colonies used in research and education
Source: PLoS One. 2024 May 21;19(5):e0287088. doi: 10.1371/journal.pone.0287088 (PMC11108223; doi:10.1371/journal.pone.0287088)
Supplement: Attachment — Submitted filename: Response to Reviewers.pdf [file pone.0287088.s009.pdf]

Dear editor, dear reviewers,

We would like to thank both reviewers for taking the time to go through our manuscript and provide useful comments to improve its quality. We worked out all comments and we see that the paper has improved. We hope that the manuscript is now suitable for publication in PLOS ONE.

Reviewer 1

1.1 The paper contains the bill of materials well described and the promising resulting imaging. However, it is not much scientific. I would expect to see in a paper like this how the equipment was designed, a computational mathematical model, some analysis, maybe some optimization. However, the paper looks like a datasheet of a product with instructions on how to build it.

We agree with reviewer 1 that not much information was given on the design process. We added sections addressing some of the key points during development, as well as results from previous iterations of the device (e.g. Appendix, Fig. S2). Reviewer 1 also commented that the submitted manuscript is not much scientific. We think that this partly concerns the unusual structure of the manuscript, which does not strictly follow the most common approach of answering a research question with available equipment. Instead, we want to introduce new research equipment and make it available for the scientific community. Therefore, the emphasis was put on providing all necessary information to build that design, making it look like the "datasheet (…) with building instructions" it is. We have no computational mathematical model but believe that the results are nonetheless useful for the research community. We have seen similar publications in PLOS One and therefore decided to submit to this journal.

1.2 I suggest not using acronyms in the title, even well-known ones like NIR.

We agree with reviewer 1 and have changed the title accordingly.

Reviewer 2

2 (…) this reviewer thinks this may fit better into one of the chemical education journals.

It was hard to categorize this manuscript because of its multi-disciplinary nature. We have considered the possibility to submit to an educational journal. However, we have no significant qualitative data from the participating schools and want to stress that the imager is indeed vital for everyday operations in our research group. It may be that in the future, we will collect more data from participating schools and publish that data set separately, in collaboration with education researchers. We have noticed similar, multi-disciplinary articles spanning equipment development, research and education published in PLOS ONE and found it best suitable for our submission.

2.1 The conclusion that the device is capable of detecting colonies as small as 0.5 mm is not supported by the data presented. Ideally, proper analysis of the detection limits of the device should be included.

Initially we believed that the scale bar in figure 4 would provide sufficient evidence that the imager records fluorescent colonies in that size range. The scale bar was done accurately with Image J using a known reference length (the length of the white label on the Petri dish). However, we now added an additional figure with more pictures taken with the device, including a set with an included ruler (Fig. S4).

The difficulty in a "proper analysis of the detection limit" is, that the detection limit is likely not only dependent on the size, but also on the number and brightness of fluorescent molecules in the cells, the total number of cells as well as cell density in the colony, all of which is very hard to analyze or predict. Therefore, artificial fluorescent samples with clear dimensions are unsuitable to assess the detection limit of fluorescent AAPB colonies. However, we hope that the new figure gives better insights into the capabilities of the device.

2.2 While demonstrating the applicability of the device in different settings is a good approach to highlight it accessibility, none of the applications presented discuss robustness and accuracy of the device in collecting emitted fluorescence. This has to be addressed using, for example, one of the standard fluorescent dyes at different concentrations across few different devices.

We have considered a lot about how to make a comparison that makes sense. While we agree that it would be nice to show fluorophores with the imager, there are several difficulties regarding their uses.

1. The amount of fluorescence emitted and captured by the camera does not only depend on fluorophore concentration, but also on the dimensions and properties of the imaged object. A high cone will result in a different fluorescence intensity than a flat droplet. Additionally, measuring suspended molecules will differ from measuring colonies. In our opinion there is no use in assessing the capabilities to detect fluorescent colonies by using samples of different nature.

2. To our knowledge there is no standard fluorophore which would be excited in the UV wavelength and emits in the NIR wavelength, similar to how AAPBs are detected by us. For example, using suspended Bacteriochlorophyll *a* as a test would be unsuitable as its fluorescence properties are different when extracted compared to being incorporated in the light harvesting complex inside a cell (fluorescence maximum at 780-800 nm in organic solution and 890-910 nm in LH1 complexes; employed excitation bandpass filter 880 nm ± 8 nm (FWHM = 70 ± 8 nm)). Changing light sources and excitation filters to match common fluorophores will unavoidably change the performance of the imager and make it unsuitable for an evaluation of its AAPB recording capabilities. Also, the camera sensors´ sensitivity will differ at different wavelength.

Comparing the same sample across different devices proves to be difficult. Imaging devices and spectrometers can be tweaked in numerous ways making a comparison difficult. Increase in sensor gain (iso), acquisition time or excitation intensity will affect the result. There is no standard device or method that would do the same AAPB colony identification as NIRis, which is why we cannot compare it to a standard method.

Concerning the robustness of the device, we believe that the amount of Petri dishes that have been imaged so far, in combination with the amount of successfully isolated AAPBs, give a hint on how robust our system is. The referenced pre-print of a separate work from our group (Nissinen et al., 2023) provides a documented excerpt which provides more detailed analysis of the isolates identified with NIRis. Additionally, previous work demonstrated that all AAPB colonies detected with the device showed a BChl *a* absorbance peak when imaged with a hyperspectral camera (Franz, 2022). This information was added to the manuscript.

2.3 Authors need to comment on the need to switch to other imaging devices (for example DSLR in Fig 5) in order to get clearer images.

The use of a DSLR simply provides cleaner, more vibrant colours and better detail than the 25€ raspberry pi camera. The white light images taken with NIRis are still necessary and suitable for the

location of fluorescent signals on a sample plate. It is however not suitable for the aesthetic presentation of agar art, which benefits from a better and more expensive camera set-up, including larger diffused lighting than it would be possible in our device. We have added some remarks in the manuscript to clarify this matter.
