# Supplementary material for: NIRis: A low-cost, versatile imaging system for near-infrared fluorescence detection of phototrophic cell colonies used in research and education
Source: PLoS One. 2024 May 21;19(5):e0287088. doi: 10.1371/journal.pone.0287088 (PMC11108223; doi:10.1371/journal.pone.0287088)
Supplement: Attachment — Submitted filename: Response_to_Reviewers.pdf [file pone.0287088.s010.pdf]

Dear editor, dear reviewers,

We would like to thank both reviewers for taking the time to go through our revised manuscript and provide useful comments to further improve its quality. We addressed all comments and think that the paper has improved. We hope that the manuscript is now suitable for publication in PLOS ONE. Below are the individual responses to each comment.

Reviewer #2: Revised manuscript does address some of the previously raised comments, but the manuscript may need few changes before publication.

1- **Can you add scale bars (or dimensions to all figures)**

Scalebars have been now added individually to each of the images taken with the NIRis device.

2- **In order to proper assess the detection limit of the system, you may be able to present colonies/cells in a better way. For example, by defining the borders of each colony and adding approximate dimensions (particularly for figures 4 and 5)**

We have now included measurements for selected colonies to provide more accurate data on the diameter of observable colonies. The measurements were done accurately with Fiji image software using a known distance to set the scale and subsequently measure diameters in each image individually.

3- **While manuscript claims that the device has been in use in 7 different high schools in Finland, it did not include any supporting information on its applicability in schools. Ideally this may include some data form students-lead projects or at least describe how this has been integrated within the program/curriculum.**

We have now included extensive information on the use of NIRis in the curriculum of participating schools, based on the teacher´s information and experiences.
